# The ELITE Portal: A FAIR Data Resource For Healthy Aging Over The Life Span

**DOI:** 10.1093/geroni/igaf122.2304

**Published:** 2025-12-31

**Authors:** Milan Vu

**Affiliations:** Sage Bionetworks, Seattle, Washington, United States

## Abstract

Exceptional longevity (EL) is a rare phenotype characterized by an extended health span and sustained physiological function. Various domain-specific factors contribute to EL, influencing the maintenance of key physiological systems (e.g., respiratory, cardiovascular, immune) and functional domains (e.g., mobility, cognition). Studying the impacts of protective genetic variants and cellular mechanisms associated with EL may facilitate the identification of novel therapeutic targets that may replicate their beneficial effects. The Exceptional Longevity Translational Resources (ELITE) Portal (eliteportal.synapse.org) is a new, open access repository for disseminating data and other research resources from translational longevity research projects. The portal supports diverse data types including genetic, transcriptomic, epigenetic, proteomic, metabolomic, and phenotypic data from longitudinal human cohort studies and cross-species comparative biology studies of tens- to hundreds- of nonhuman species; data from longevity-enhancing intervention studies in mouse and cell models; access to web applications and software tools to support exploration of EL-related research outcomes; and a catalog of publications associated with the National Institute on Aging (NIA)-funded translational longevity research projects. The portal also features integration with the external Trusted Research Environment (TRE) CAVATICA and is poised to support future integrations with additional data resources like Terra. All resources hosted in the ELITE Portal are distributed under FAIR (Findable, Accessible, Interoperable, and Reusable) principles. The ELITE Portal is funded by the NIA 5U24AG078753 and 2U24AG061340.

